# A Genome-Wide Over-Expression Screen Identifies Genes Involved in Phagocytosis in the Human Protozoan Parasite, *Entamoeba histolytica*

**DOI:** 10.1371/journal.pone.0043025

**Published:** 2012-08-14

**Authors:** Ada V. King, Brenda H. Welter, Amrita B. Koushik, Lindsay N. Gordon, Lesly A. Temesvari

**Affiliations:** 1 Department of Biological Sciences, Clemson University, Clemson, South Carolina, United States of America; 2 Department of Genetics and Biochemistry, Clemson University, Clemson, South Carolina, United States of America; University of Oklahoma Health Sciences Center, United States of America

## Abstract

Functional genomics and forward genetics seek to assign function to all known genes in a genome. *Entamoeba histolytica* is a protozoan parasite for which forward genetics approaches have not been extensively applied. It is the causative agent of amoebic dysentery and liver abscess, and infection is prevalent in developing countries that cannot prevent its fecal-oral spread. It is responsible for considerable global morbidity and mortality. Given that the *E. histolytica* genome has been sequenced, it should be possible to apply genomic approaches to discover gene function. We used a genome-wide over-expression screen to uncover genes regulating an important virulence function of *E. histolytica*, namely phagocytosis. We developed an episomal *E. histolytica* cDNA over-expression library, transfected the collection of plasmids into trophozoites, and applied a high-throughput screen to identify phagocytosis mutants in the population of over-expressing cells. The screen was based on the phagocytic uptake of human red blood cells loaded with the metabolic toxin, tubercidin. Expression plasmids were isolated from trophozoites that survived exposure to tubercidin-charged erythrocytes (phagocytosis mutants), and the cDNAs were sequenced. We isolated the gene encoding profilin, a well-characterized cytoskeleton-regulating protein with a known role in phagocytosis. This supports the validity of our approach. Furthermore, we assigned a phagocytic role to several genes not previously known to function in this manner. To our knowledge, this is the first genome-wide forward genetics screen to be applied to this pathogen. The study demonstrates the power of forward genetics in revealing genes regulating virulence in *E. histolytica*. In addition, the study validates an *E. histolytica* cDNA over-expression library as a valuable tool for functional genomics.

## Introduction

Functional genomics is a branch of molecular biology that seeks to assign roles to all known genes in a genome. It encompasses a wide variety of techniques which may be classified as reverse or forward genetics. Reverse genetics begins by mutating a gene of interest. Subsequently, mechanisms of gene action may be uncovered by observing the ensuing phenotypic changes. On the other hand, forward genetics techniques begin with a mutant phenotype of interest; altered genes contributing to this phenotype are then identified. With the completion of genome sequences for many organisms, it is now possible to perform genome-wide reverse or forward genetics screens to uncover novel genes regulating known cellular processes.

Genome-wide over-expression screens represent one of many types of forward genetics approaches (reviewed in [1]). This method uses low- to high-throughput selection schemes to identify and select for certain phenotypes in populations of cells over-expressing all or a subset of genes from the genome. Genes that have been identified by over-expression screens include, but are not limited to, those that regulate the action of small-molecules [2], the cell cycle [3], [4], oxidative stress-induced damage [5], endocytic vesicle trafficking [6], and development [7]. Thus, it is clear that forward genetics and over-expression screens represent a powerful approach for gene discovery.

*Entamoeba histolytica* is a protozoan parasite for which forward genetics approaches have not been extensively applied. It is causative agent of amoebic dysentery and liver abscess (reviewed in [8]) and is prevalent in developing countries that cannot prevent its fecal-oral spread. The pathogen is responsible for considerable global morbidity and mortality. Infection is acquired by ingestion of the cyst form of the parasite. Excystation occurs in the small intestine; released amoeboid trophozoites move to, and colonize, the bowel lumen. Here, the pathogen acquires nutrients via phagocytosis of colonic bacteria, host cells including red blood cells (RBCs), and host cell debris. The ability of the pathogen to carry out phagocytosis has been correlated to its virulence potential. For example, phagocytosis-deficient mutants of *E. histolytica* exhibit reduced pathogenicity *in vitro* and *in vivo* [9], [10], and a non-invasive *Entamoeba* species, *E. dispar*, exhibits low rates of phagocytosis [11].

Phagocytosis is a complex cellular activity that relies on the coordinated regulation of signal transduction, cytoskeletal rearrangement, and membrane remodeling. Given the importance of phagocytosis to *E. histolytica* virulence, the identification of proteins that directly or indirectly regulate this process has been the focus of a considerable research effort (reviewed in [12], [13]). Proteomic analyses have identified numerous proteins associated with purified phagosomes of *E. histolytica* [13]–[17]. However, discerning the exact functions of these genes in phagoctyosis requires additional experimentation.

The genome of *E. histolytica* has been sequenced [18]. Thus, it may be possible to apply functional genomics approaches to identify genes regulating virulence functions, such as phagocytosis. Here, we report on the application of an over-expression screen to discover genes regulating phagocytic trafficking. Specifically, we adapted a high-throughput scheme [19] that relied on a loss-of-function phenotype (reduced phagocytosis) to uncover genes that, when over-expressed, negatively regulate phagocytosis. We uncovered the gene encoding profilin, a well-characterized cytoskeleton-regulating protein with a known role in phagocytosis [20]. This supports the validity of our approach. Furthermore, we assigned a phagocytic role to several genes not previously known to function in this manner. This study demonstrates that is possible to use forward genetics for gene discovery in this important global pathogen.

## Methods

### Strains and Culture Conditions

*Entamoeba histolytica* trophozoites (strain HM-1:IMSS) were cultured axenically in TYI-S-33 media [21] in 15 ml glass screw cap tubes at 37°C. The generation of *E. histolytica* cell lines conditionally over-expressing green fluorescent protein (GFP) or a GFP-tagged pleckstrin homology domain from mammalian Bruton’s tyrosine kinase (GFP-PH^Btk^) is described elsewhere [22]. These two transgenic cell lines were maintained in TYI-S-33 supplemented with 6 µg/ml G418 and 15 µg/ml hygromycin. GFP or GFP-PH^Btk^ expression was induced by the addition of 5 µg/ml tetracycline to the culture medium 24 hours prior to performing assays.

### Construction of a cDNA Expression Library and Transfection

An *E. histolytica* cDNA library was constructed using Invitrogen Custom Services (Invitrogen, Carlsbad, CA). Briefly, RNA was isolated from approximately 2.4×10^7^ trophozoites using Trizol® Reagent (Invitrogen) according to the manufacturer’s protocol. Poly(A)^+^-RNA was selected from 2 mg total RNA and used to generate a primary, uncut, 7-fold normalized cDNA library, directionally cloned into the Gateway® compatible entry vector, pENTR™222 (f1-). Proprietary 5′ cap-binding technology was used to select and enrich for full-length clones. The final titer of the amplified library was 3.9×10^6^ colony-forming units/ml. Twenty-four random clones, with a minimum of 20 different genes, were analyzed for the presence of inserts. All 24 clones had inserts with an average size of 1.1 kb.

The LR Clonase system (Invitrogen) was used to transfer cDNA inserts from pENTR™222 to pAH-DEST [23], a Gateway-compatible *E. histolytica* expression vector (kind gift from C.A. Gilchrist and W.A. Petri, Jr., Dept. of Medicine, Division of Infectious Diseases and International Health, University of Virginia, Charlottesville, VA), using manufacturer’s instructions. This plasmid is episomal in nature [24] and confers hygromycin-resistance to transfectants. Expression from this plasmid is driven by upstream ferredoxin regulatory sequences. The cDNA library in pAH-DEST was transfected into HM1:IMSS trophozoites using standard protocols [22], [25]. Transfectants were maintained by the addition of 23 µg/ml hygromycin to the medium.

### Development of a Screen for Phagocytosis Mutants

Human red blood cells (hRBCs) (US Biological Inc., Swampscott, MA) were washed three times with phosphate-buffered saline (PBS) and resuspended to 5% (vol/vol) in PBS. Subsequently, erythrocytes were exposed to tubercidin (7-deazaadenosine) (500 µg/ml) (Sigma, St. Louis, MO) or an equivalent volume of DMSO (diluent) for 1 hour at 37°C. The treated erythrocytes were washed five times in PBS and resuspended to 0.5% (vol/vol) in serum-free trophozoite medium. Log-phase trophozoites (1.5×10^5^ cells/ml) were exposed to the tubercidin-loaded hRBCs for 30 minutes at 37°C at a ratio of ∼100 hRBCs:1 amoeba. This ratio represents saturation of erythrophagocytic receptors in *E. histolytica* [26]. After incubation, the trophozoites and hRBCs were collected by centrifugation (500×*g*, 1 minute) and un-internalized hRBCs were hypotonically lysed by the addition of distilled water to the pellets. The trophozoites were washed with PBS, resuspended in TYI-S-33 medium, and incubated at 37°C. Trophozoites were allowed to recover for 1 hour, after which, the selection scheme was repeated once (for a total of 2 treatments) or twice (for a total of 3 treatments). Finally, trophozoites were incubated at 37°C for 24 to 48 hours. Cell viability was measured by microscopy with Trypan Blue exclusion.

### Isolation of Episomes from Survivors and Sequencing

Total DNA was isolated from unselected control cells and cells that survived selection using the Wizard Genomic DNA Purification Kit (Promega, Madison, WI). The isolated DNA was introduced into *Escherichia coli* (XL10-GOLD, Stratagene, La Jolla, CA) by standard transformation protocols resulting in ampicillin-resistant bacterial colonies. Bacterial colonies were randomly chosen from each condition (control or tubercidin-selection), transferred to the wells of 96-well plates containing LB medium supplemented with ampicillin (100 µg/ml) and glycerol (50% vol/vol) and stored at −80°C prior to automated sequencing at the Arizona State University BioDesign Institute (Tempe, AZ).

### Analysis of Expression by Quantitative Real-Time PCR

*E. histolytica* cells were transfected with the expression plasmids encoding H644 or EhLimA using standard methods [22], [25]. The transfectants were grown to mid log phase (3×10^5^ cells/ml). Total RNA was isolated with Trizol® Reagent, and 2 µg RNA was treated with DNAase I for 30 minutes at 37°C. cDNA was synthesized with oligo-dT and Superscript III reverse transcriptase (Invitrogen) at 50°C for 2 hours.

Expression of H644 or EhLimA was measured by quantitative real-time PCR (qPCR) using the RT^2^ SYBR Green Fluor qPCR Mastermix (SABiosciences, Frederick, MD) and the appropriate primers for EhLimA (forward primer 5′-TGGTGATTCTAGTGAACGCCGTGA-3′; reverse primer 5′-TCATGTACCTCTTCTTCGTGAACT-3′) and H644 (forward primer 5′-GCTACCGTTGCTGAAGATAGAGCAAGAC-3′; reverse primer 5′-GCAATGCTATCTCTGAATGGAGCAG-3′). Levels of cDNA were normalized using the small subunit ribosomal RNA (ssRNA) gene (Accession No. X61116) as a housekeeping gene [27] (forward primer 5′-AGGCGCGTAAATTACCCACTTTCG-3′; reverse primer 5′-CACCAGACTTGCCCTCCAATTGAT-3′) and ratios were calculated using the Pfaffl method [28]. qPCR was performed using an IQ5 I-Cycler (Bio-Rad Laboratories Inc., Hercules, CA). Two independent biological replicates were evaluated. For each cDNA three technical replicates were performed and the values averaged. The efficiency of each primer pair was assessed by use of a dilution series. In all cases, efficiency values were ≥88.2% and R^2^ values were ≥0.989.

### Erythrophagocytosis Assay

Measurement of erythrophagocytosis, using human erythrocytes, was carried out according to the methods of Voigt *et al.* [29] with modifications. Erythrocytes were washed 3 times by centrifugation with serum-free amoeba culture medium to eliminate endogenous serum and then resuspended in serum-free amoeba culture medium. *E. histolytica* cells were washed with serum-free amoeba culture medium. Amoebae (2×10^5^) were incubated with hRBCs (ratio of hRBCs:amoebae; 100∶1) at 37°C in 0.2 ml serum-free amoeba culture medium for 10 minutes. The amoebae and erythrocytes were then centrifuged and resuspended in 2 ml cold sterile water to lyse non-ingested hRBCs. After a second centrifugation step, the pellet contained only amoebae with internalized hRBCs. The pellet was washed with PBS and resuspended in 1 ml concentrated formic acid. Samples were measured against a formic acid blank with a µQuant spectrophotometer plate reader (Bio-Tek, Winooski, VT) at 405 nm. Phagocytosis was reported as the percent of uptake by un-transfected control cells, which was arbitrarily set to 100%.

### Fluid Phase Endoctyosis Assay

*E. histolytica* cells, suspended in serum-free medium, were exposed to 5 mg/ml fluorescein isothiocyanate-dextran (FITC-dextran; 40 kDa) (Sigma, St. Louis, MO) in serum-free medium and incubated for 30 minutes at 37°C. The amoebae were collected by centrifugation, washed twice in ice-cold PBS, and lysed by the addition of 2.5% (vol/vol) Triton X-100 in PBS. Total fluorescence of the samples was measured using an FLx800 microplate fluorescence reader (Bio-Tek) with excitation and emission wavelengths of 485 nm and 528 nm, respectively. Endocytosis was reported as the percent of uptake by un-transfected control cells, which was arbitrarily set to 100%.

### Cycloheximide-Sensitivity Assay

*E. histolytica* trophozoites (3×10^4^ cells) were seeded into 13 mL of TYI-S-33 supplemented with 100 nM cycloheximide or an equivalent volume of PBS (diluent). The cells were incubated at 37°C for 48 hours after which viability was assessed by microscopy with Trypan-blue exclusion.

### Statistical and *In Silico* Analyses

All values are given as a mean ± standard deviation (S.D.) of at least 3 trials. To compare means, statistical analyses were performed using GraphPad Instat® V.3 with an unpaired *t*-test, Welch corrected (two-tail *P*-value). To compare the composition of the gene set obtained before and after selection we used a modified χ2 test of independence, known as a G-test [30]. In all cases, *P*-values less than 0.05 were considered statistically significant and were denoted by a single asterisk (*). *P*-values less than 0.01 or 0.001 were considered highly statistically significant and were denoted by two (**) or three (***) asterisks, respectively. To identify structural and functional residues and domains, the predicted amino acid sequences of the H644 and EhLimA proteins were analyzed using ExPASy ScanPROSITE [31]. Cartoon renderings of the H644 and EhLimA proteins were generated using the MyDomains-Image Creator in ExPASy PROSITE.

## Results

### Development of a Screen to Enrich for Phagocytosis Mutants

Tubercidin is a cytotoxic adenosine analog isolated from *Streptomyces tubercidus.* Its mode of action is related to its ability to inhibit glycolysis [32] and/or intercalate into DNA [33]. Tubercidin readily permeates erythrocytes whereupon it is converted to a membrane impermeant phosphorylated form [34]. Tubercidin-loaded erythrocytes have been used to isolate phagocytosis mutants from a population of chemically mutagenized macrophages [19]. To determine if tubercidin-charged erythrocytes could be used to identify *E. histolytica* phagocytosis mutants, we assessed the toxicity of this selection agent on trophozoites. Amoebae were exposed to toxin-loaded hRBCs and the viability of the trophozoites was assessed.

**Figure 1:**
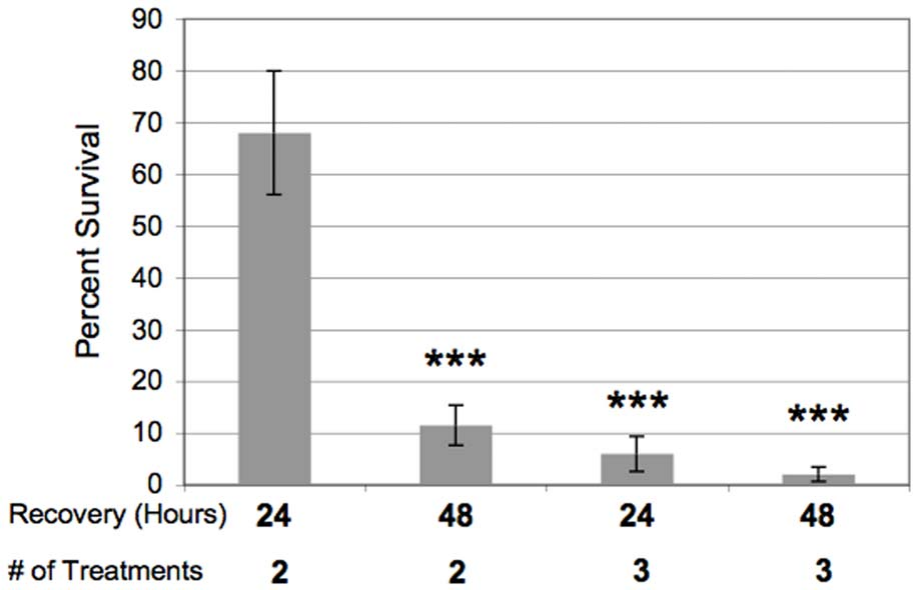
Tubercidin-loaded erythrocytes are toxic to *E. histolytica*. Trophozoites were exposed to tubercidin-loaded erythrocytes two or three times (# of Treatments) and viability was assessed 24 hours or 48 hours after treatment. The data are reported as a percent of the starting number of viable amoebae before treatment. The data represent the mean ± S.D of ≥3 trials (***P<0.001). Three treatments of *E. histolytica* cells with tubercidin-charged hRBCs resulted in the death of nearly 100% of the cells 48 hours after application of selection.

Three treatments of *E. histolytica* cells with tubercidin-charged hRBCs resulted in the death of nearly 100% of the cells 48 hours after application of selection (Fig. 1). This level of toxicity suggested that this selection regimen may be useful in isolating phagoctyosis mutants from a population of *E. histolytica* cells. Single (data not shown) and double exposures (Fig. 1) to tubercidin-loaded hRBCs were not sufficient to achieve near 100% death. For example, 2 treatments with tubercidin-loaded hRBCs resulted in 68±12% and 11.5±3.9% viability 24 and 48 hours after selection, respectively (Fig. 1). Multiple rounds of exposure to toxin-loaded erythrocytes were also required to achieve high levels of cell death in macrophages [19]. As a control, we also exposed *E. histolytica* cells to hRBCs treated with the tubercidin vehicle, DMSO. This did not decrease viability of the trophozoites suggesting the killing was specific to tubercidin (data not shown).

### Validation of the Screen Using a Known Phagocytosis Mutant

**Figure 2:**
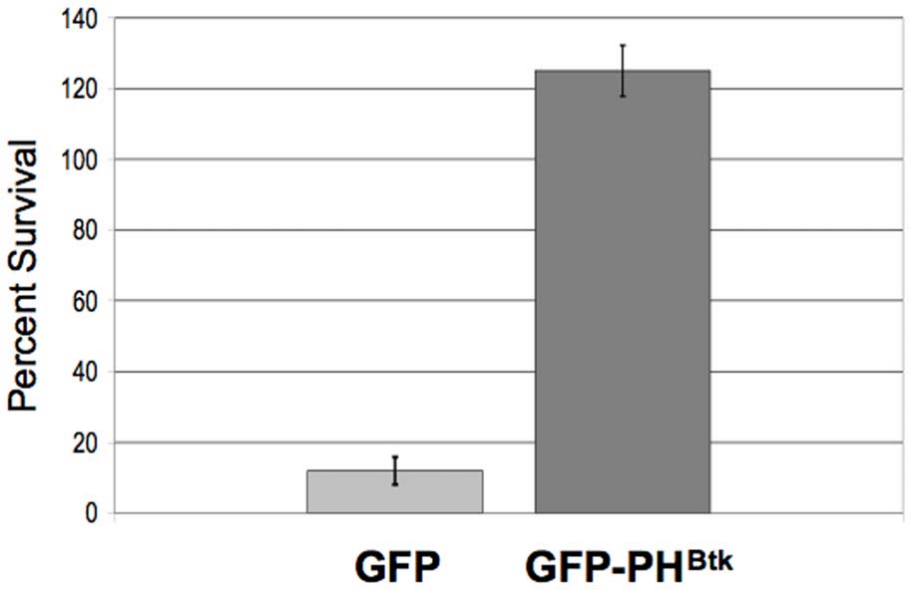
Tubercidin-loaded erythrocytes are less toxic to an *E. histolytica* cell line with a phagocytosis defect. Trophozoites over-expressing a GFP-tagged version of a pleckstrin homology (PH) domain from mammalian Bruton’s tyrosine kinase (GFP-PH^Btk^), which exhibit reduced phagocytosis [22] or a control cell line over-expressing GFP alone (GFP) were exposed to tubercidin-loaded erythrocytes three times and viability was assessed 48 hours after treatment. The data are reported as a percent of the starting number of viable amoebae before treatment. The data represent the mean ± S.D of 3 trials. The phagocytic mutant was insensitive to treatment with tubercidin-charged hRBCs as evidenced by >100% survival (growth) in the presence of selection. This indicates that the selection scheme may be used to enrich for phagocytosis mutants from a population of cells.

To validate the selection scheme, we assessed the toxicity of tubercidin-loaded hRBCs on an *E. histolytica* cell line with a known defect in phagocytosis. We used a transgenic cell line expressing a GFP-tagged version of a pleckstrin homology (PH) domain from mammalian Bruton’s tyrosine kinase (GFP-PH^Btk^) [22]. This mutant exhibits a 69% reduction in phagocytosis when compared to a control GFP-expressing cell line [22]. However, the GFP-PH^Btk^-expressing cell line displays normal fluid-phase endocytosis [22]. Tubercidin-loaded hRBCs were less toxic to the cell line expressing GFP-PH^Btk^ as compared to a cell line expressing GFP alone (Fig. 2). This indicates that our selection scheme may be used to enrich for phagocytosis mutants from a population of cells. Since the mutant showed a high level of survival in the presence of selection, it suggests that there is negligible free residual tubercidin being taken up by fluid-phase endocytosis, an alternative endocytic pathway that is normal in this mutant.

### Screening with Tubercidin-Charged Erythrocytes Identifies Proteins That May Function in Phagocytosis

*E. histolytica* cells were transformed with a cDNA over-expression library constructed in the episomal pAH-DEST expression vector [23]. Expression from this vector is constitutively by sequences derived from the upstream region of the *E. histolytica* ferredoxin gene [24]. Given the high level of toxicity associated with 3 rounds of selection, the transgenic population of cells was exposed to tubercidin-loaded hRBCs 3 times as described above. After a 48 hour recovery period, surviving trophozoites were lysed and over-expressed cDNAs were identified by purification of episomes, amplification of episomes in *E. coli*, and sequencing. As a control, episomes were also isolated and sequenced from the transgenic population of trophozoites that was not subjected to selection.

Table 1 shows the identity and frequency of cDNAs isolated from trophozoites that survived selection as compared to those recovered randomly from a population of over-expressing cells not subjected to selection. To determine if the set of genes enriched after selection was significantly different from that in the control population, we used a modified χ2 test of independence, known as a G-test. We determined that the set of genes isolated by selection was extremely statistically different from the set of genes isolated randomly from the control unselected population (*P* = 4.7×10^−7^). This statistical analysis shows strong evidence for a non-fortuitous enrichment of specific genes.

**Table 1:** Isolation of cDNAs from *E. histolytica* over-expressors without (Control) and with (Selected) selection with tubercidin-charged erythrocytes.

GenInfo Identifier (GI) Number	Gene Name	Number of Isolates (Control)	Number of Isolates (Selected)
183232640	Hypothetical protein	6	55
183234305	Ribosomal protein L10	1	8
1070154	Profilin	0	6
183233949	Ribosomal protein L7	5	6
67483282	EhLimA	0	4
183230765	Ribosomal protein L9	6	3
158992	SREHP	1	2
183230551	V-ATP synthase, subunit E	1	2
183232834	Ribsomal protein L13	0	2
67469498	Rho-activating protein	1	2
183232074	Hypothetical protein	1	1
183230481	Ribosomal protein S9	1	1
67479568	Histone H2b, putative	0	1
67481134	Ribosomal protein S25	4	1
183232219	Hypothetical protein	9	0
67479580	Alcohol dehydrogenase	7	0
732691	Alcohol dehydrogenase 3	6	0
183235741	Ribosomal protein L15	5	0
183233758	Ribosomal protein L13	4	0
183231176	Enolase, putative	4	0
183232711	Ribosomal protein S14	4	0
183232055	Ribosomal protein L17	3	0
183230950	C2-domain protein	3	0
183233207	Actin-binding protein	2	0
183233272	Serine protease inhibitor	2	0
25989681	GTPase, putative	2	0
183233845	Ribosomal protein L14	2	0
183231806	Ribosomal protein L11	1	0
183231333	Ribosomal protein L4	1	0
183230617	Ribosomal protein L23	1	0
183232892	Ribosomal protein S7	1	0
67466875	Ribosomal protein S25	1	0
183236693	Serine-rich protein	1	0
25989677	HSP70-like protein	1	0
183230634	Hypothetical protein	1	0
183234340	Ras family GTPase	1	0
**Total**		**89**	**90**

We expected to enrich for genes with a known function in phagocytosis. We also predicted that we would identify novel genes that regulate this cellular function. The cDNAs encoding a hypothetical protein (GI:183232640), ribosomal protein L10 (GI:183234305), profilin (GI:1070154), and a LIM zinc finger domain-containing protein (EhLimA; GI:67483282) were the most highly enriched by selection with tubercidin-loaded erythrocytes.

The cDNA encoding profilin was isolated from 6.3% of bacterial clones that had been transfected with episomes isolated from trophozoite survivors. Profilin is an abundant actin monomer-binding protein that plays a role in the regulation of cytoskeleton restructuring (reviewed in [35]). Profilin also has a known role in phagocytosis in other systems [20], [36], [37]. Given the established role of profilin in phagocytosis, we did not conduct additional studies with this gene. However, the isolation of profilin by our screen supports the authenticity of the selection protocol. A cDNA encoding ribosomal protein L10 was also isolated by the screen with high frequency (8.5% of sequenced clones). L10 may be an authentic regulator of phagocytosis or it may be represent a false positive. Since over-expression of ribosomal proteins may have profound global and non-specific effects on protein synthesis, this gene was also not considered further for the purposes of this study. Many ribosomal proteins were also isolated in the absence of selection (Table 1).

**Figure 3:**
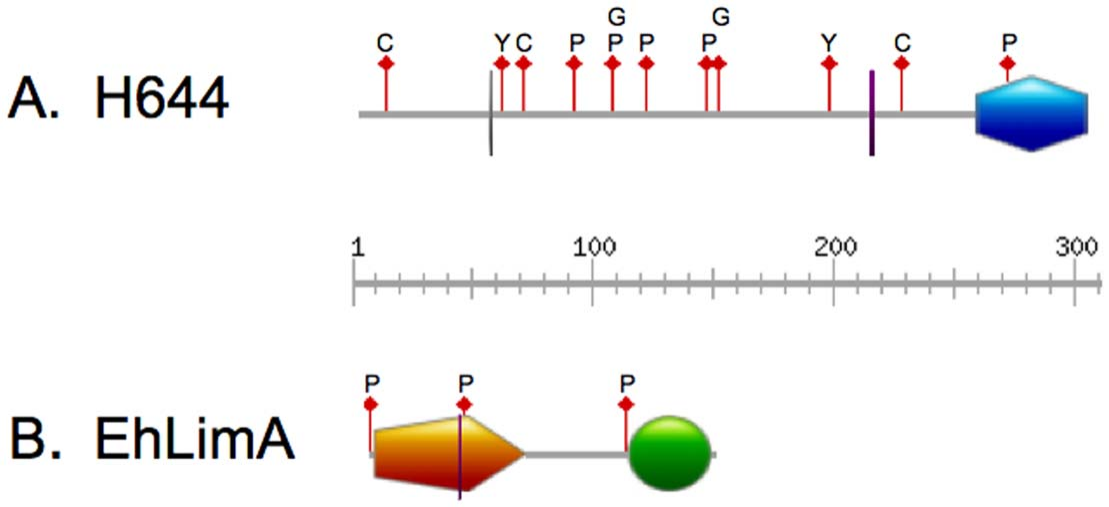
Schematic of domains found in the hypothetical protein H644 and EhLimA. A. H644 is a hypothetical protein with a postulated molecular weight of 33.8 kDa (304 amino acids). It is predicted to have a lysine-rich region (blue hexagon), a glycosylation site (gray vertical line), a N-myristoylation site (purple vertical line), 3 casein kinase II phosphorylation sites (red flags labeled C), 5 protein kinase C phosphorylation sites (red flags labeled P), 2 tyrosine kinase phosphorylation sites (red flags labeled Y), and 2 cAMP/cGMP-dependant protein kinase phosphorylation sites (red flags labeled G). B. EhLimA has a molecular weight of 15.9 kDa (145 amino acids). It has an N-terminal LIM domain (orange pentagon), a C-terminal glutamic acid-rich region (green circle), 3 protein kinase C phosphorylation sites (red flags labeled P), and a N-myristoylation site (purple vertical line).

The cDNA encoding a hypothetical protein was the most highly enriched of the cDNAs and was present in 58.5% of the bacterial clones that had been transfected with episomes isolated from trophozoite survivors. The corresponding protein, that we named H644, is predicted to be 304 amino acids long (33.8 kDa) and to possess a C-terminal lysine rich region, and casein kinase II, tyrosine kinase, protein kinase C, cAMP-dependant, and cGMP-dependent phosphorylation sites (Fig. 3A). These phosphorylation sites are consistent with a role in cell signaling. The protein also possesses putative N-glycosylation and myristoylation sites (Fig. 3A).

The cDNA encoding a LIM zinc finger-containing protein, also known as EhLimA [38], was present in 4.2% of bacterial clones that had been transfected with the episomes isolated from trophozoite survivors. This protein is predicted to be 145 amino acids long (15.9 kDa). Like other members of the LIM family (reviewed in [39]), the protein has two putative contiguous zinc finger domains (LIM domains) separated by a two-amino acid hydrophobic linker (Fig. 3B). EhLimA also possesses a glutamic acid-rich region, a PKC phosphorylation site, and an N-myristoylation site (Fig. 3B).

### Authentication of H644 and EhLimA as Negative Regulators of Phagocytosis

**Figure 4:**
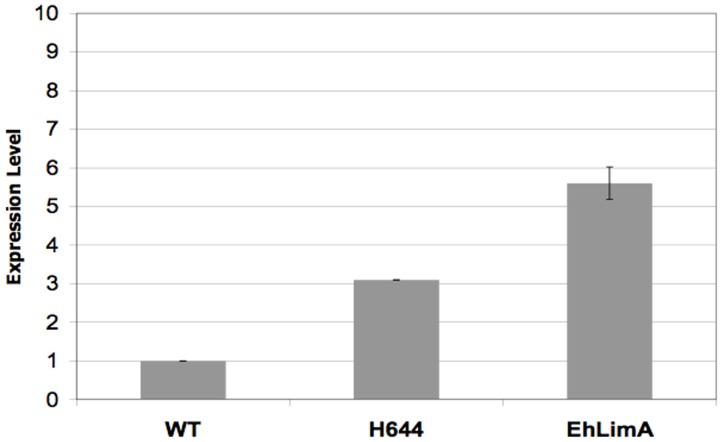
qPCR confirms over-expression of H644 and EhLimA. RNA from untransfected (wild-type; WT) log phase *E. histolytica* trophozoites or from trophozoites transfected with an expression vector encoding H644 or EhLimA was used for qPCR analysis of expression. The ssRNA gene was used as a loading control. H644 and EhLimA were expressed at approximately 3.1 (±0.0)- and 5.6 (±0.4)-fold, respectively, over wild-type levels.

Expression vectors encoding H644 or EhLimA, isolated from the original screen, were transfected into wild-type trophozoites to construct 2^nd^ generation cell lines uniformly over-expressing these proteins. Over-expression of H644 or EhLimA transcripts was confirmed by qPCR. H644 or EhLimA transcripts exhibited a 3.1 (±0.0)- and 5.6 (±0.4)-fold higher abundance, respectively, in the 2^nd^ generation transgenic cell lines as compared to parental controls (Fig. 4).

**Figure 5:**
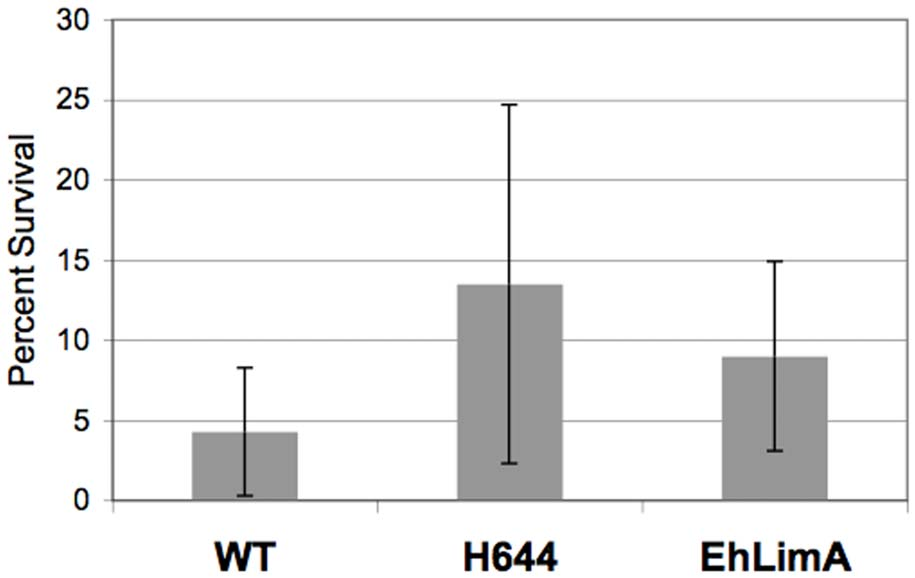
Tubercidin-loaded erythrocytes are less toxic to *E. histolytic* cells over-expressing H644 or EhLimA. Transgenic trophozoites were exposed to tubercidin-loaded erythrocytes (3 treatments) and viability was assessed 48 hours after treatments. The data are reported as a percent of the starting number of viable amoebae before treatment. The data represent the mean ± S.D of 3 trials. *E. histolytica* transgenic cells exposed to tubercidin-charged hRBCs displayed increased survival as compared to untransfected wild-type (WT) cells.

To authenticate the identification of EhLimA or H644 as negative regulators of phagocytosis, we exposed the 2^nd^ generation transgenic cell lines to 3 rounds of selection with tubercidin-charged hRBCs. After a 48 hour recovery period we re-assessed toxicity of the selecting agent. These cell lines exhibited increased survival in the presence of cytotoxic erythrocytes as compared to control cells (Fig. 5) suggesting that their isolation during the original scheme was valid. Although the increase in survival was not statistically significant, this degree of viability in the presence of toxin may have been sufficient to confer enrichment of these over-expressors during the original screen. Though we observed >3-fold increase in expression of EhLimA or H644 in the 2^nd^ generation cell lines, we do not know the level of over-expression in the initial isolates since they were not cloned. Thus, we cannot rule out a gene dosage effect as over-expression may have been higher in the original mutants that survived selection.

**Figure 6:**
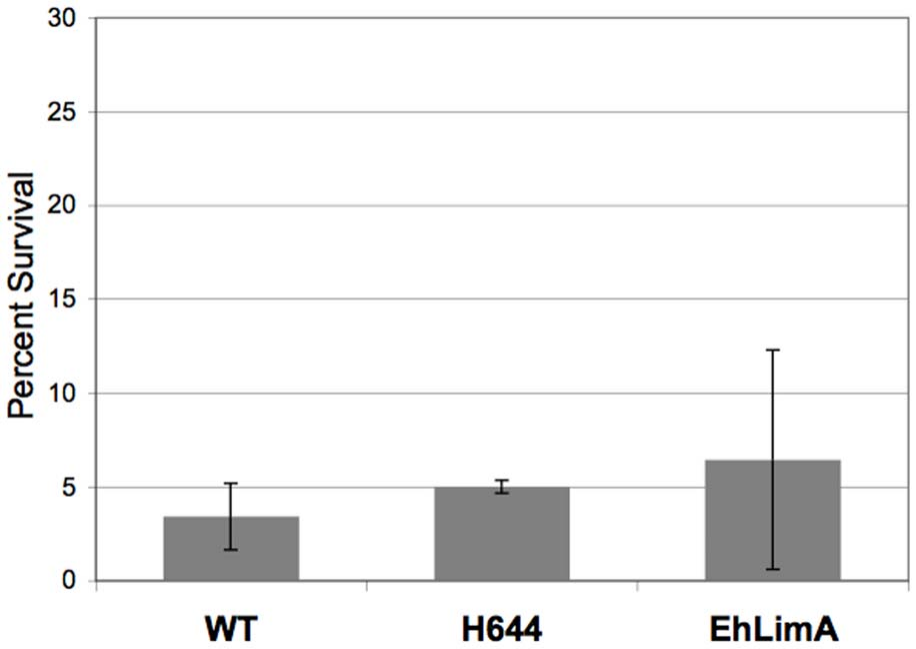
Cycloheximide is toxic to *E. histolytica* cells over-expressing H644 or EhLimA. Transgenic trophozoites were exposed to 100 nM cycloheximide for 48 hours after which viability was assessed. The data are reported as a percent of untreated control amoebae. The data represent the mean ± S.D of 3 trials. Cycloheximide was toxic to both untransfected wild-type (WT) amoebae and *E. histolytica* transgenic cells. Therefore, cells over-expressing H644 or EhLimA do not exhibit a multidrug resistance phenotype.

If H644 or EhLimA function as cell surface pumps, or if they enhance the activity of cell surface pumps, general resistance to small molecules (i.e., a multidrug resistance phenotype) may be conferred. If this were the case, the isolation of H644 or EhLimA over-expressors would be considered a false positive outcome of the screen. To address this, the specificity of the response to tubercidin-loaded hRBCs was tested by assaying the effect of an unrelated small molecule, cycloheximide, on the H644- and EhLimA-expressing cell lines. The transgenic cell lines were exposed to cycloheximide and viability was determined. When compared to control cells, neither transgenic cell line exhibited increased survival in the presence of cycloheximide (Fig. 6). This indicated that isolation of H644 or EhLimA during the original screen was not due to a multidrug resistance phenotype characterized by non-specific extrusion of small molecules.

**Figure 7:**
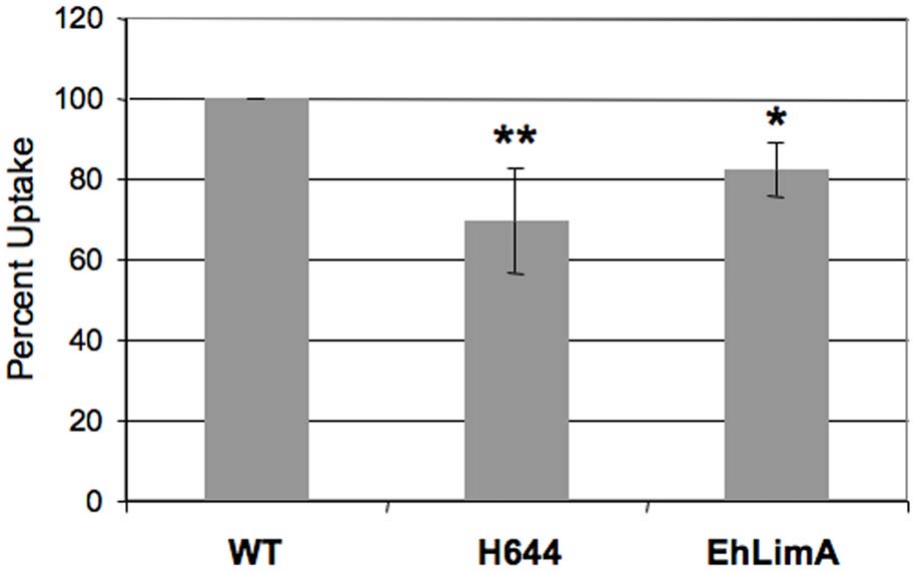
Phagocytosis is reduced in *E. histolytica* cells over-expressing H644 or EhLimA. Wild-type (WT) or transgenic cells were incubated with erythrocytes (hRBC:amoeba ratio; 100∶1) for 10 minutes, lysed, and spectrophotometrically analyzed for internalized heme at 405 nm. The data represent the mean ± S.D. of 4 experiments (**P<0.01; *P<0.05). Amoebae over-expressing H644 or EhLimA exhibit reduced phagocytosis of hRBCs.

**Figure 8:**
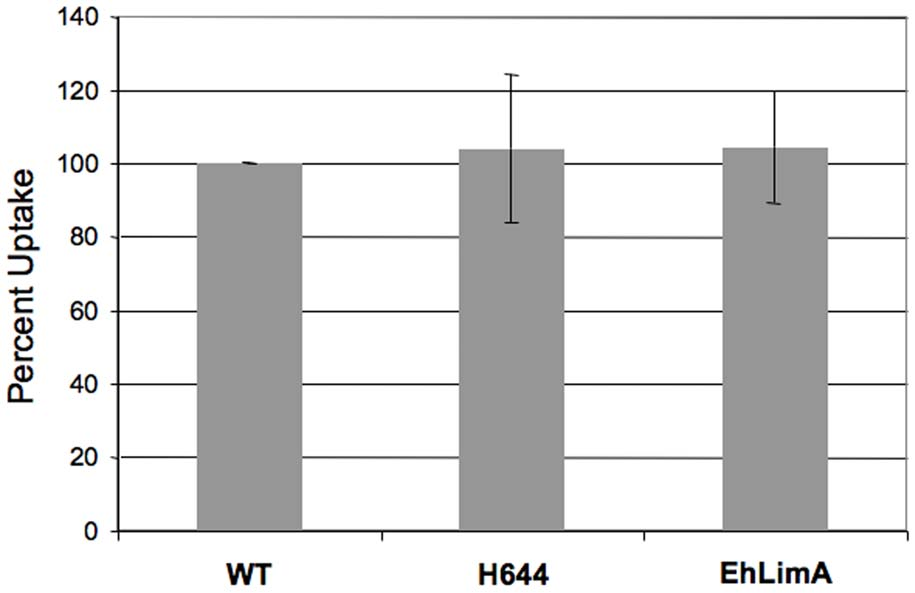
Fluid-phase endocytosis is not reduced in *E. histolytica* cells over-expressing H644 or EhLimA. Wild-type (WT) or transgenic cells were incubated with the fluid-phase marker, FITC-dextran for 30 minutes, lysed, and analyzed for internalized FITC-dextran using spectrofluorimetry. The data represent the mean ± S.D. of ≥3 experiments. Amoebae over-expressing H644 or EhLimA exhibit normal uptake of fluid-phase marker suggesting that these cell lines do not have wide-spread defects in endocytosis.

To confirm that the isolation of H644 and EhLimA over-expressors from a mixed population was the result of a phagocytosis defect, we performed the definitive experiment by measuring uptake of erythrocytes in the 2^nd^ generation cell lines. Control and mutant cell lines were exposed to hRBCs for 10 minutes, after which extracellular erythrocytes were lysed hypotonically with distilled water and the level of ingested heme was measured by spectrophotometry. Erythrophagocytosis in H644 and EhLimA over-expressing cell lines was inhibited by approximately 31% and 18%, respectively, when compared to parental control cells (Fig. 7). This supports a role for these proteins in the regulation of phagocytosis. To determine if the transgenic cell lines expressing H644 or EhLimA possessed a general endocytic defect, we also measured uptake of a fluid-phase marker, FITC-dextran. Importantly, uptake of this marker was not affected by over-expression of H644 or EhLimA (Fig. 8). Thus, H644 and EhLimA appear to specifically regulate the phagocytosis of erythrocytes.

## Discussion

In this study we have shown that genes regulating important virulence functions in *E. histolytica* can be identified using a functional genomics approach. Since phagocytosis is an important virulence determinant, it was chosen as the focus of the screen. However, our goal was not to study phagocytosis *per se*, but to determine the utility of over-expression screens for gene discovery in *E. histolytica*. To the best of our knowledge, this is the first forward genetics over-expression screen applied to *E. histolytica*. Specifically, we adapted a high-throughput protocol to enrich for phagocytosis mutants in a mixed population of over-expressing cells and identified a LIM-domain-containing protein (EhLimA) and a hypothetical protein (H644) as putative negative regulators of phagocytosis.

*E. histolytica* phagocytoses apoptotic and non-apoptotic host cells by different routes and receptors [12]. Although we did not observe any obvious changes in morphology of tubercidin-charged hRBCs by light microscopy, we cannot eliminate the possibility that tubercidin induced an apoptosis-like event in these host cells. Never-the-less, EhLimA- and H644-overexpressors were isolated in the screen using tubercidin-loaded hRBCs (apoptotic?) and also exhibited reduced phagocytosis of unloaded hRBCs (non-apoptotic). Thus, we predict that EhLimA and H644 are more general regulators of phagocytosis participating in uptake of either apoptotic or non-apoptotic host erythrocytes.

We also identified the actin-binding protein, profilin, using our screen. At high concentrations, profilin prevents the polymerization of actin [35]. Therefore, it is reasonable to hypothesize that profilin over-expression would inhibit phagocytosis, and it was not surprising that this protein was identified by our screen. In support of this, profilin over-expression reduces endocytic uptake in murine cells [36] and in a non-pathogenic amoeba, *Dictyostelium discoideum* [37]. In addition, *Drosophila melanogaster* [20] profilin-null mutants exhibit increased phagocytosis. Given the established role of profilin in phagocytosis [20], [36], [37], the enrichment of a profilin over-expressor validates the reliability of the screen.

A ribosomal protein (L10) was also highly enriched in our screen. L10 may be an authentic regulator of phagocytosis or it may represent a false positive. It has recently been shown that under some conditions, phagosomes fuse with auto-phagosomes. Since auto-phagosomes originate from invaginations of the endoplasmic reticulum [40], an organelle rich in ribosomes and ribosomal proteins, it would not be surprising to detect ribosomal proteins in phagosomes. Furthermore, phagosome–autophagosome fusion during mycobacterial infection is characterized by the transport of cytosolic ribosomal proteins into the lumen of these organelles and the subsequent degradation of these proteins into mycobactericidal peptides [41]. Thus, L10 may be an authentic phagosome protein.

Since our screen was based on a functional assay, we predict that we would not have enriched for phagosomal cargo proteins unless these cargo proteins also specifically regulate phagosome function. A study of *E. histolytica* ribosomal protein L10 [42] revealed that this protein was localized to the nucleus and that its over-expression suppressed growth by 60% and disrupted the AP-1/c-Jun transcription factor complex [42]. Suppression of growth is likely coupled with reduced metabolic requirements and, by extension, with reduced sensitivity to metabolic toxins. Therefore, an L10-over-expressor could have been enriched in our screen by a slow-growth phenotype conferring reduced sensitivity to tubercidin, a metabolic poison. In either case, it remains to be seen if L10 authentically controls phagocytosis of erythrocytes in *E. histolytica.*

In the current study, a loss-of-function phenotype (reduced phagocytosis), coupled with viability, formed the basis of the screen. The current screen was based on a previous scheme in which tubercidin-charged erythrocytes were used to enrich for phagocytosis variants from a population of macrophages chemically mutagenized with nitrosoguanidine (NG) [19]. Although it was not possible to identify specific macrophage genes that were altered by NG-treatment, 40 stable clones were isolated and characterized and it was found that the majority of these cell lines were authentic phagocytosis mutants. Only one of the 40 clones was a false positive clone that likely survived selection by non-specific extrusion of the toxin (i.e., multidrug resistance). This illustrates the robustness of a selection protocol that uses toxin-charged erythrocytes to identify mutants defective in erythrophagocytosis. We have combined this robustness with newer technology that allows for the specific identification of mutated (over-expressed) genes to identify novel phagocytosis-regulators in *E. histolytica*.

Loss-of-function screens have been used in other systems to identify genes regulating a variety of cellular functions. For example, in *Saccharomyces cerevisiae*, loss-of-function screens have uncovered genes that regulate nutrient acquisition and metabolism [43]–[49], sensitivity to various forms of irradiation [50]–[53], sensitivity to anti-fungal agents [54], [55], and resistance to various other small molecules, peptides, and toxins [56]–[60]. In *Caenorhabditis elegans,* lethality was the readout in several large-scale genome-wide screens [61]–[64]. In human HeLa cells, viability was scored in a screen to identify genes, specifically kinases, involved in apoptosis [65]. Our data suggest that over-expression combined with a loss-of-function phenotype may also be valuable in gaining insight into *E. histolytica* virulence.

Although transfection efficiency in *E. histolytica* is low, the possibility that members of the library of over-expressing cells harbored more than one plasmid was a caveat of our original screen. Thus, confirming the “hits” was critical in our study. We used two approaches to confirm the roles of EhLimA and H644 in phagocytosis. First, we created 2^nd^ generation *E. histolytica* cell lines over-expressing these proteins, and we repeated the original assay using tubercidin-charged erythrocytes. Second, we quantified erythrophagocytosis in the new transgenic cell lines. Both lines of experimentation confirmed the authenticity of these hits as each 2^nd^ generation cell line exhibited increased survival in the presence of the selection agent and decreased phagocytosis. The cell line over-expressing H644 showed a higher level of survival in the presence of selection and a more pronounced phagocytosis defect. Thus, it was not surprising that H644 was isolated more frequently than EhLimA. Since over-expression of EhLimA or H644 alone (2^nd^ generation cell lines) led to reduced phagocytosis, it was unlikely that original over-expressors harbored additional plasmids that were necessary for conferring the phenotype.

Several proteomic analyses of purified phagosomes from *E. histolytica* have been carried out. Our work complements these analyses. Marion *et al*. [14] conducted proteomic analysis of purified *E. histolytica* phagosomes using liquid chromatography and tandem mass spectroscopy (LC-MS/MS). Both profilin and EhLimA were also identified as members of the *E. histolytica* phagosome proteome. Thus, our functional screen extends our knowledge by specifically identifying EhLimA and profilin as negative regulators of phagocytosis in *E. histolytica*.

EhLimA has been the subject of another study in *E. histolytica* [38]. In the previous report, the authors demonstrated that EhLimA was localized to the parasite cell surface, interacted with the cytoskeleton through its N-terminal LIM domain, and resided in lipid rafts. In addition, the authors showed that neither transcriptional silencing nor over-expression of EhLimA affected growth or morphology of trophozoites. Our study advances the understanding of the role of EhLimA in cellular functions, specifically in phagocytosis.

EhLimA exhibits a high degree of homology with DdLimE, a LIM-domain-containing protein from *D. discoideum* [66], [67]. Although a role for DdLimE in phagocytosis has not been established, several other *D. discoideum* LIM family proteins, LimF and ChLim, have well-defined and opposing roles in this process [68]. For example, over-expression of LimF or loss of ChLim increases the rate of phagocytosis, whereas loss of LimF or over-expression of ChLim inhibits phagocytosis. EhLimA was also identified as a protein that was over-expressed in an avirulent strain of *E. histolytica* that was incapable of inducing liver abscess in experimentally infected rodents [69]. Erythrophagocytosis was not inhibited in this avirulent strain; however, there were numerous other concurrent genetic changes in this subspecies. Together, these genetic changes likely contributed to the phenotype of this strain.

Currently, it remains to be seen if inhibition of phagocytosis is a direct or indirect effect of over-expression of the hypothetical protein, H644. Although hypothetical proteins were highly represented in the *E. histolytica* phagosome proteome [15], [16], H644 was not identified as a phagosome-interacting protein in these analyses. Given the significant number of putative phosphorylation sites in H644, this protein is predicted to have a role in signal transduction. Indirect inhibition of phagocytosis could occur by over-expression of a protein that is part of, or communicates with, a signaling relay that regulates phagocytosis. Nonetheless, the identification of H644 as a negative regulator of phagocytosis in the current study illustrates one of the advantages of functional genomics screens over proteomic analysis of purified organelles. Here, we have identified a protein that may not necessarily interact with phagosomes but exhibits a role in regulating phagocytosis.

Other functional genomics screens using this library of over-expressing trophozoites can be envisioned. For example, screens to identify a different class of endocytic mutants may be conducted by applying fluid phase toxins to the transgenic population and characterizing the episomes isolated from survivors. Employing fluorescent phagocytic markers and fluorescence-associated cell sorting (FACS) could be used to isolate mutants with enhanced phagocytosis instead of decreased phagocytosis. A high-throughput centrifugation-based adhesion assay has recently been described for mammalian cells [70]. The adaptation of such an assay to examine adhesion in the population of *E. histolytica* over-expressors would represent a screen that could provide insight into another important virulence function, namely parasite-host interaction.

Insight into drug resistance may be gained by selecting for metronidazole-resistant mutants from the population of over-expressing cells. Drug resistance is not prevalent in *E. histolytica*. However, occasional reports of metronidazole failures that cannot be attributed to a lack of patient compliance, suggest the possibility for the development of clinical resistance [71]–[73]. Identification of over-expressed genes in metronidazole-resistant isolates may paint a picture of the propensity of this pathogen to acquire resistance to small-molecules. Screening of small-molecule libraries for anti-microbial agents is becoming increasingly popular [74], [75]. If small molecule inhibitors of *E. histolytica* growth are identified, then the targets of such inhibitors might be rapidly discovered by subsequently screening the library of over-expressing transformants for resistant mutants.

Systematic functional genomics screens have become standard practice in assigning gene function and have accelerated our understanding of cell biology in both mammalian and non-mammalian systems. Although our screen was not exhaustive, we have demonstrated the power of functional genomics in revealing genes regulating a virulence function in *E. histolytica*. Our results also validate the *E. histolytica* cDNA over-expression library as a valuable tool for functional genomics. As other high-throughput screens are developed for *E. histolytica*, their application to the library of over-expressing transformants will undoubtedly provide rapid insight into *E. histolytica* pathogenicity and may reveal novel targets for the development of drugs or vaccines.
